# Ethyl 2-diethyl­amino-4-oxo-3,5-diphenyl-4,5-dihydro-3*H*-pyrrolo­[3,2-*d*]pyrimidine-7-carboxyl­ate

**DOI:** 10.1107/S1600536811052883

**Published:** 2011-12-14

**Authors:** Hai-Tao Gao, Li Li, Fang Ye, Yang-Gen Hu

**Affiliations:** a Institute of Medicinal Chemistry, Hubei University of Medicine, Shiyan 442000, People’s Republic of China; b The Library of Hubei University of Medicine, Shiyan 442000, People’s Republic of China; c Department of Pharmacy, Taihe Hospital of Hubei University of Medicine, Shiyan 442000, People’s Republic of China

## Abstract

In the title compound, C_25_H_26_N_4_O_3_, the two fused pyrrolo­[3,2-*d*]pyrimidine rings form a dihedral angle of 3.7 (2)°. The two substituent phenyl rings are twisted with respect to the pyrrole and pyrimidine rings, making dihedral angles of 57.2 (2) and 69.0 (2)°, respectively. The ethyl and eth­oxy groups are disordered over two positions; the site occupancies are 0.53 (1) and 0.47 (1) for ethyl, and 0.63 (1) and 0.37 (1) for eth­oxy. The crystal packing features C—H⋯O hydrogen bonds.

## Related literature

For the synthesis, see: Hu *et al.* (2006 ▶, 2007 ▶, 2010 ▶). For related structures, see: He *et al.* (2007*a* ▶,*b* ▶); Ma *et al.* (2009 ▶); Zeng & Yan (2008 ▶).
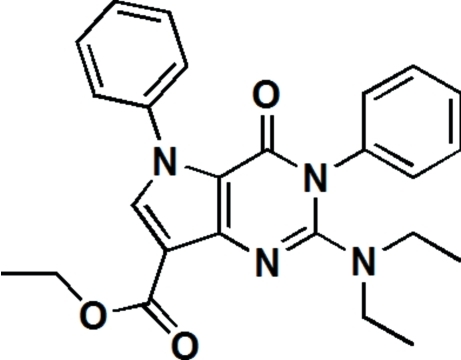


## Experimental

### 

#### Crystal data

C_25_H_26_N_4_O_3_
*M* *_r_* = 430.50
Monoclinic, 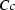

*a* = 19.481 (2) Å
*b* = 12.0745 (13) Å
*c* = 10.4393 (11) Å
β = 115.006 (2)°
*V* = 2225.4 (4) Å^3^
*Z* = 4
Mo *K*α radiation
μ = 0.09 mm^−1^
*T* = 298 K
0.20 × 0.10 × 0.10 mm

#### Data collection

Bruker SMART 4K CCD area-detector diffractometer
Absorption correction: multi-scan (*SADABS*; Bruker, 2002 ▶) *T*_min_ = 0.973, *T*_max_ = 0.991
7050 measured reflections
2192 independent reflections
1846 reflections with *I* > 2σ(*I*)
*R*_int_ = 0.050

#### Refinement

*R*[*F* ^2^ > 2σ(*F* ^2^)] = 0.046
*wR*(*F* ^2^) = 0.104
*S* = 1.00
2192 reflections
332 parameters
12 restraints
H-atom parameters constrained
Δρ_max_ = 0.15 e Å^−3^
Δρ_min_ = −0.19 e Å^−3^

### 

Data collection: *SMART* (Bruker, 2002 ▶); cell refinement: *SAINT* (Bruker, 2002 ▶); data reduction: *SAINT*; program(s) used to solve structure: *SHELXS97* (Sheldrick, 2008 ▶); program(s) used to refine structure: *SHELXL97* (Sheldrick, 2008 ▶); molecular graphics: *PLATON* (Spek, 2009) ▶; software used to prepare material for publication: *SHELXTL* (Sheldrick, 2008 ▶).

## Supplementary Material

Crystal structure: contains datablock(s) I, global. DOI: 10.1107/S1600536811052883/yk2034sup1.cif

Structure factors: contains datablock(s) I. DOI: 10.1107/S1600536811052883/yk2034Isup2.hkl

Supplementary material file. DOI: 10.1107/S1600536811052883/yk2034Isup3.cml

Additional supplementary materials:  crystallographic information; 3D view; checkCIF report

## Figures and Tables

**Table 1:** Hydrogen-bond geometry (Å, °)

*D*—H⋯*A*	*D*—H	H⋯*A*	*D*⋯*A*	*D*—H⋯*A*
C6—H6⋯O1^i^	0.93	2.50	3.320 (5)	147
C19—H19*B*⋯O1^ii^	0.96	2.59	3.545 (8)	175
C25—H25⋯O2^iii^	0.93	2.60	3.295 (5)	132

Symmetry codes: (i) 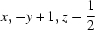; (ii) 
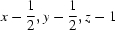
; (iii) 
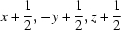
.

**Fig. 1.**
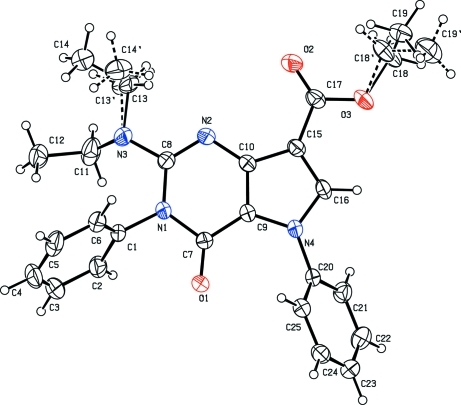
The molecular structure of the title compound showing the atom-labeling scheme with displacement ellipsoids drawn at the 30% probability level.

## supplementary crystallographic information

### Comment

As a part of our ongoing work in the preparation of derivatives of heterocyclic compounds (Hu *et al.*, 2006, 2007, 2010; He *et al.*, 2007a,b; Ma *et al.* 2009; Zeng *et al.*, 2008), we have synthesized and structurally characterized the title compound (Fig. 1). In the molecule, the two fused rings of pyrrolo[3,2-*d*]pyrimidine form a dihedral angle of 3.7 (2)°. The attached two phenyl rings are twisted with respect to the heterocyclic pyrrolo[3,2-*d*]pyrimidine system, making dihedral angles of 56.8 (2)° and 69.3 (2)°, respectively. The ethyl group (C13—C14) is disordered over two sites with occupancies that refined to 0.53 (1) and 0.47 (1); the ethoxy group (C18—C19) is also disordered over two sites with occupancies 0.63 (1) and 0.37 (1). The crystal packing is stabilized by intermolecular C—H···O hydrogen bonds. There are no π-π interactions.

### Experimental

The title compound was obtained in good yield *via* aza-Wittig reaction. Crystals suitable for single-crystal X-ray diffraction were obtained by recrystallization from a mixture of ethanol and dichloromethane (1:1 *v*/*v*) at room temperature.

### Refinement

All H atoms were located in difference maps and treated as riding atoms with C—H = 0.93 Å, *U*_iso_=1.2*U*_eq_ (C) for C*sp*^2^, C—H = 0.97 Å, *U*_iso_ = 1.2*U*_eq_ (C) for CH_2_, C—H = 0.96 Å, *U*_iso_ = 1.5*U*_eq_ (C) for CH_3_. N—H = 0.86 Å, *U*_iso_ = 1.2*U*_eq_ (N) for NH.

### Crystal data

**Table:**

C_25_H_26_N_4_O_3_	*F*(000) = 912
*M**_r_* = 430.50	*D*_x_ = 1.285 Mg m^−^^3^
Monoclinic, *C**c*	Mo *K*α radiation, λ = 0.71073 Å
Hall symbol: C -2yc	Cell parameters from 2386 reflections
*a* = 19.481 (2) Å	θ = 2.3–23.4°
*b* = 12.0745 (13) Å	µ = 0.09 mm^−^^1^
*c* = 10.4393 (11) Å	*T* = 298 K
β = 115.006 (2)°	Block, colourless
*V* = 2225.4 (4) Å^3^	0.20 × 0.10 × 0.10 mm
*Z* = 4	

### Data collection

**Table:**

Bruker SMART 4K CCD area-detector diffractometer	2192 independent reflections
Radiation source: fine-focus sealed tube	1846 reflections with *I* > 2σ(*I*)
graphite	*R*_int_ = 0.050
φ and ω scans	θ_max_ = 26.0°, θ_min_ = 2.0°
Absorption correction: multi-scan (*SADABS*; Bruker, 2002)	*h* = −24→24
*T*_min_ = 0.973, *T*_max_ = 0.991	*k* = −14→14
7050 measured reflections	*l* = −10→12

### Refinement

**Table:**

Refinement on *F*^2^	Secondary atom site location: difference Fourier map
Least-squares matrix: full	Hydrogen site location: inferred from neighbouring sites
*R*[*F*^2^ > 2σ(*F*^2^)] = 0.046	H-atom parameters constrained
*wR*(*F*^2^) = 0.104	*w* = 1/[σ^2^(*F*_o_^2^) + (0.063*P*)^2^] where *P* = (*F*_o_^2^ + 2*F*_c_^2^)/3
*S* = 1.00	(Δ/σ)_max_ < 0.001
2192 reflections	Δρ_max_ = 0.15 e Å^−^^3^
332 parameters	Δρ_min_ = −0.19 e Å^−^^3^
12 restraints	Absolute structure: no
Primary atom site location: structure-invariant direct methods	

### Special details

**Table:**

Geometry. All e.s.d.'s (except the e.s.d. in the dihedral angle between two l.s. planes) are estimated using the full covariance matrix. The cell e.s.d.'s are taken into account individually in the estimation of e.s.d.'s in distances, angles and torsion angles; correlations between e.s.d.'s in cell parameters are only used when they are defined by crystal symmetry. An approximate (isotropic) treatment of cell e.s.d.'s is used for estimating e.s.d.'s involving l.s. planes.
Refinement. Refinement of *F*^2^ against ALL reflections. The weighted *R*-factor *wR* and goodness of fit *S* are based on *F*^2^, conventional *R*-factors *R* are based on *F*, with *F* set to zero for negative *F*^2^. The threshold expression of *F*^2^ > σ(*F*^2^) is used only for calculating *R*-factors(gt) *etc*. and is not relevant to the choice of reflections for refinement. *R*-factors based on *F*^2^ are statistically about twice as large as those based on *F*, and *R*- factors based on ALL data will be even larger.

### Fractional atomic coordinates and isotropic or equivalent isotropic displacement parameters (Å^2^)

**Table:**

	*x*	*y*	*z*	*U*_iso_*/*U*_eq_	Occ. (<1)
C1	0.35411 (19)	0.5896 (2)	0.3254 (4)	0.0403 (7)	
C2	0.3739 (3)	0.6278 (3)	0.4599 (4)	0.0603 (10)	
H2	0.3554	0.5935	0.5188	0.072*	
C3	0.4218 (3)	0.7183 (4)	0.5069 (5)	0.0839 (15)	
H3	0.4357	0.7452	0.5980	0.101*	
C4	0.4489 (3)	0.7682 (3)	0.4199 (7)	0.0839 (15)	
H4	0.4802	0.8300	0.4513	0.101*	
C5	0.4306 (2)	0.7280 (3)	0.2885 (6)	0.0697 (13)	
H5	0.4500	0.7615	0.2306	0.084*	
C6	0.3834 (2)	0.6381 (3)	0.2401 (4)	0.0489 (8)	
H6	0.3713	0.6102	0.1501	0.059*	
C7	0.33929 (19)	0.3910 (3)	0.3331 (3)	0.0401 (8)	
C8	0.23552 (19)	0.5019 (3)	0.1557 (4)	0.0481 (8)	
C9	0.29509 (19)	0.3005 (2)	0.2527 (3)	0.0398 (7)	
C10	0.22868 (18)	0.3156 (3)	0.1325 (3)	0.0415 (7)	
C11	0.1974 (3)	0.6797 (3)	0.2194 (6)	0.0808 (14)	
H11A	0.2229	0.6464	0.3122	0.097*	
H11B	0.1438	0.6830	0.1972	0.097*	
C12	0.2268 (3)	0.7972 (3)	0.2256 (6)	0.0738 (12)	
H12A	0.2789	0.7949	0.2407	0.111*	
H12B	0.2225	0.8365	0.3019	0.111*	
H12C	0.1974	0.8343	0.1381	0.111*	
C13	0.1572 (6)	0.6089 (8)	−0.0445 (10)	0.063 (3)	0.53
H13A	0.1804	0.5621	−0.0907	0.075*	0.53
H13B	0.1085	0.5767	−0.0613	0.075*	0.53
C14	0.1442 (5)	0.7208 (7)	−0.1108 (8)	0.071 (2)	0.53
H14A	0.1194	0.7671	−0.0686	0.107*	0.53
H14B	0.1130	0.7143	−0.2103	0.107*	0.53
H14C	0.1920	0.7532	−0.0962	0.107*	0.53
C13'	0.1387 (5)	0.6277 (11)	−0.0102 (11)	0.060 (4)	0.47
H13C	0.1144	0.6960	−0.0032	0.072*	0.47
H13D	0.1033	0.5668	−0.0282	0.072*	0.47
C14'	0.1678 (6)	0.6352 (11)	−0.1238 (9)	0.080 (3)	0.47
H14D	0.2101	0.6851	−0.0936	0.120*	0.47
H14E	0.1283	0.6622	−0.2098	0.120*	0.47
H14F	0.1835	0.5632	−0.1398	0.120*	0.47
C15	0.19836 (18)	0.2084 (3)	0.0816 (4)	0.0438 (8)	
C16	0.2478 (2)	0.1342 (3)	0.1758 (4)	0.0460 (8)	
H16	0.2418	0.0577	0.1696	0.055*	
C17	0.1284 (2)	0.1791 (3)	−0.0417 (4)	0.0497 (8)	
C18	0.0502 (6)	0.0198 (12)	−0.1557 (11)	0.066 (4)	0.63
H18A	0.0079	0.0694	−0.1744	0.079*	0.63
H18B	0.0403	−0.0499	−0.1203	0.079*	0.63
C19	0.0625 (5)	0.0017 (8)	−0.2874 (9)	0.069 (2)	0.63
H19A	0.0705	0.0718	−0.3224	0.103*	0.63
H19B	0.0187	−0.0336	−0.3581	0.103*	0.63
H19C	0.1061	−0.0445	−0.2656	0.103*	0.63
C18'	0.0571 (10)	0.0377 (18)	−0.1873 (18)	0.066 (7)	0.37
H18C	0.0465	0.0971	−0.2556	0.079*	0.37
H18D	0.0122	0.0258	−0.1708	0.079*	0.37
C19'	0.0759 (11)	−0.0666 (16)	−0.245 (2)	0.097 (5)	0.37
H19D	0.1168	−0.0523	−0.2708	0.146*	0.37
H19E	0.0322	−0.0906	−0.3266	0.146*	0.37
H19F	0.0906	−0.1234	−0.1739	0.146*	0.37
C20	0.3654 (2)	0.1325 (2)	0.3965 (3)	0.0411 (7)	
C21	0.3453 (2)	0.0630 (3)	0.4797 (4)	0.0541 (9)	
H21	0.2946	0.0540	0.4616	0.065*	
C22	0.4006 (3)	0.0071 (3)	0.5897 (5)	0.0684 (12)	
H22	0.3872	−0.0402	0.6457	0.082*	
C23	0.4755 (3)	0.0207 (3)	0.6173 (4)	0.0635 (11)	
H23	0.5129	−0.0167	0.6924	0.076*	
C24	0.4952 (2)	0.0900 (3)	0.5332 (4)	0.0558 (9)	
H24	0.5459	0.0984	0.5508	0.067*	
C25	0.4401 (2)	0.1468 (3)	0.4232 (4)	0.0477 (8)	
H25	0.4535	0.1945	0.3676	0.057*	
N1	0.30567 (16)	0.4938 (2)	0.2734 (3)	0.0427 (6)	
N2	0.19752 (16)	0.4179 (2)	0.0831 (3)	0.0499 (7)	
N3	0.20773 (19)	0.6083 (2)	0.1163 (4)	0.0717 (11)	
N4	0.30646 (16)	0.18742 (19)	0.2789 (3)	0.0411 (6)	
O1	0.39918 (14)	0.38737 (18)	0.4370 (3)	0.0535 (6)	
O2	0.08390 (15)	0.2436 (2)	−0.1197 (3)	0.0663 (8)	
O3	0.12107 (15)	0.0691 (2)	−0.0540 (3)	0.0713 (8)	

### Atomic displacement parameters (Å^2^)

**Table:**

	*U*^11^	*U*^22^	*U*^33^	*U*^12^	*U*^13^	*U*^23^
C1	0.0400 (17)	0.0294 (14)	0.0459 (19)	−0.0009 (13)	0.0126 (15)	−0.0004 (13)
C2	0.086 (3)	0.0422 (18)	0.049 (2)	−0.0030 (18)	0.024 (2)	−0.0038 (17)
C3	0.107 (4)	0.051 (2)	0.060 (3)	−0.002 (3)	0.002 (3)	−0.019 (2)
C4	0.062 (3)	0.046 (2)	0.117 (4)	−0.018 (2)	0.012 (3)	−0.015 (3)
C5	0.054 (2)	0.0420 (19)	0.116 (4)	0.0014 (18)	0.039 (3)	0.015 (2)
C6	0.050 (2)	0.0412 (17)	0.059 (2)	0.0023 (15)	0.0267 (18)	0.0026 (15)
C7	0.0405 (19)	0.0384 (16)	0.0391 (19)	−0.0017 (13)	0.0146 (17)	0.0007 (13)
C8	0.041 (2)	0.0399 (18)	0.054 (2)	−0.0010 (15)	0.0115 (18)	0.0056 (15)
C9	0.0412 (18)	0.0346 (15)	0.0420 (18)	−0.0043 (14)	0.0159 (15)	−0.0034 (13)
C10	0.0368 (18)	0.0405 (16)	0.0429 (18)	−0.0054 (14)	0.0126 (15)	−0.0032 (14)
C11	0.060 (3)	0.048 (2)	0.141 (5)	0.0094 (19)	0.048 (3)	0.006 (3)
C12	0.071 (3)	0.047 (2)	0.096 (3)	0.009 (2)	0.028 (2)	−0.005 (2)
C13	0.034 (5)	0.049 (6)	0.082 (9)	0.013 (4)	0.001 (5)	0.011 (5)
C14	0.071 (6)	0.067 (5)	0.067 (5)	0.005 (4)	0.020 (5)	0.002 (4)
C13'	0.027 (6)	0.044 (5)	0.094 (8)	0.010 (4)	0.012 (5)	0.005 (5)
C14'	0.076 (7)	0.095 (9)	0.057 (7)	0.012 (6)	0.017 (6)	0.010 (6)
C15	0.0385 (19)	0.0446 (18)	0.0441 (19)	−0.0059 (14)	0.0136 (15)	−0.0065 (15)
C16	0.0461 (19)	0.0362 (16)	0.053 (2)	−0.0108 (15)	0.0184 (16)	−0.0120 (15)
C17	0.037 (2)	0.055 (2)	0.054 (2)	−0.0072 (17)	0.0166 (17)	−0.0114 (18)
C18	0.050 (5)	0.067 (6)	0.071 (6)	−0.028 (4)	0.018 (5)	−0.018 (5)
C19	0.050 (4)	0.074 (5)	0.062 (5)	−0.006 (4)	0.004 (4)	−0.008 (4)
C18'	0.050 (9)	0.067 (11)	0.071 (12)	−0.028 (8)	0.018 (9)	−0.018 (9)
C19'	0.091 (12)	0.102 (13)	0.085 (12)	−0.015 (11)	0.024 (10)	−0.028 (10)
C20	0.0461 (19)	0.0305 (15)	0.0409 (18)	−0.0023 (14)	0.0129 (15)	−0.0044 (13)
C21	0.055 (2)	0.0455 (18)	0.063 (2)	−0.0034 (17)	0.0259 (19)	0.0050 (18)
C22	0.081 (3)	0.053 (2)	0.069 (3)	−0.001 (2)	0.029 (2)	0.017 (2)
C23	0.066 (3)	0.047 (2)	0.057 (2)	0.0078 (18)	0.007 (2)	0.0025 (18)
C24	0.044 (2)	0.051 (2)	0.061 (2)	0.0010 (16)	0.0110 (18)	−0.0144 (18)
C25	0.047 (2)	0.0423 (17)	0.049 (2)	−0.0041 (15)	0.0159 (17)	−0.0048 (15)
N1	0.0402 (15)	0.0330 (13)	0.0479 (16)	−0.0051 (12)	0.0118 (13)	−0.0016 (12)
N2	0.0387 (16)	0.0438 (16)	0.0527 (17)	−0.0048 (12)	0.0054 (14)	0.0019 (13)
N3	0.049 (2)	0.0425 (17)	0.089 (3)	0.0028 (14)	−0.0042 (19)	0.0060 (17)
N4	0.0415 (15)	0.0316 (12)	0.0454 (15)	−0.0047 (12)	0.0139 (13)	−0.0031 (12)
O1	0.0523 (15)	0.0408 (12)	0.0467 (14)	−0.0044 (11)	0.0007 (13)	−0.0021 (10)
O2	0.0453 (15)	0.0629 (16)	0.0679 (18)	0.0027 (13)	0.0018 (14)	−0.0078 (13)
O3	0.0526 (17)	0.0558 (15)	0.0760 (19)	−0.0153 (13)	−0.0016 (14)	−0.0192 (15)

### Geometric parameters (Å, °)

**Table:**

C1—C2	1.370 (5)	C13'—H13C	0.9700
C1—C6	1.375 (5)	C13'—H13D	0.9700
C1—N1	1.446 (4)	C14'—H14D	0.9600
C2—C3	1.386 (6)	C14'—H14E	0.9600
C2—H2	0.9300	C14'—H14F	0.9600
C3—C4	1.369 (8)	C15—C16	1.378 (5)
C3—H3	0.9300	C15—C17	1.469 (5)
C4—C5	1.353 (7)	C16—N4	1.355 (4)
C4—H4	0.9300	C16—H16	0.9300
C5—C6	1.374 (5)	C17—O2	1.194 (5)
C5—H5	0.9300	C17—O3	1.336 (4)
C6—H6	0.9300	C18—O3	1.466 (8)
C7—O1	1.212 (4)	C18—C19	1.509 (10)
C7—N1	1.419 (4)	C18—H18A	0.9700
C7—C9	1.424 (4)	C18—H18B	0.9700
C8—N2	1.295 (4)	C19—H19A	0.9600
C8—N3	1.387 (4)	C19—H19B	0.9600
C8—N1	1.402 (4)	C19—H19C	0.9600
C9—C10	1.382 (5)	C18'—O3	1.472 (11)
C9—N4	1.392 (4)	C18'—C19'	1.505 (11)
C10—N2	1.377 (4)	C18'—H18C	0.9700
C10—C15	1.429 (5)	C18'—H18D	0.9700
C11—N3	1.458 (6)	C19'—H19D	0.9600
C11—C12	1.521 (6)	C19'—H19E	0.9600
C11—H11A	0.9700	C19'—H19F	0.9600
C11—H11B	0.9700	C20—C25	1.373 (5)
C12—H12A	0.9600	C20—C21	1.378 (5)
C12—H12B	0.9600	C20—N4	1.440 (4)
C12—H12C	0.9600	C21—C22	1.374 (6)
C13—C14	1.490 (9)	C21—H21	0.9300
C13—N3	1.546 (9)	C22—C23	1.373 (6)
C13—H13A	0.9700	C22—H22	0.9300
C13—H13B	0.9700	C23—C24	1.378 (6)
C14—H14A	0.9600	C23—H23	0.9300
C14—H14B	0.9600	C24—C25	1.378 (5)
C14—H14C	0.9600	C24—H24	0.9300
C13'—N3	1.451 (9)	C25—H25	0.9300
C13'—C14'	1.518 (10)		
			
C2—C1—C6	120.5 (3)	C16—C15—C17	125.4 (3)
C2—C1—N1	120.7 (3)	C10—C15—C17	129.0 (3)
C6—C1—N1	118.7 (3)	N4—C16—C15	111.0 (3)
C1—C2—C3	118.9 (4)	N4—C16—H16	124.5
C1—C2—H2	120.5	C15—C16—H16	124.5
C3—C2—H2	120.5	O2—C17—O3	124.5 (3)
C4—C3—C2	120.3 (4)	O2—C17—C15	125.3 (3)
C4—C3—H3	119.9	O3—C17—C15	110.2 (3)
C2—C3—H3	119.9	O3—C18—C19	104.8 (8)
C5—C4—C3	120.3 (4)	O3—C18—H18A	110.8
C5—C4—H4	119.9	C19—C18—H18A	110.8
C3—C4—H4	119.9	O3—C18—H18B	110.8
C4—C5—C6	120.4 (4)	C19—C18—H18B	110.8
C4—C5—H5	119.8	H18A—C18—H18B	108.9
C6—C5—H5	119.8	O3—C18'—C19'	110.0 (13)
C5—C6—C1	119.6 (4)	O3—C18'—H18C	109.7
C5—C6—H6	120.2	C19'—C18'—H18C	109.7
C1—C6—H6	120.2	O3—C18'—H18D	109.7
O1—C7—N1	121.0 (3)	C19'—C18'—H18D	109.7
O1—C7—C9	127.8 (3)	H18C—C18'—H18D	108.2
N1—C7—C9	111.1 (3)	C18'—C19'—H19D	109.5
N2—C8—N3	119.8 (3)	C18'—C19'—H19E	109.5
N2—C8—N1	124.2 (3)	H19D—C19'—H19E	109.5
N3—C8—N1	116.0 (3)	C18'—C19'—H19F	109.5
C10—C9—N4	108.5 (3)	H19D—C19'—H19F	109.5
C10—C9—C7	122.3 (3)	H19E—C19'—H19F	109.5
N4—C9—C7	129.1 (3)	C25—C20—C21	120.5 (3)
N2—C10—C9	123.6 (3)	C25—C20—N4	120.7 (3)
N2—C10—C15	128.8 (3)	C21—C20—N4	118.8 (3)
C9—C10—C15	107.4 (3)	C22—C21—C20	119.7 (4)
N3—C11—C12	114.6 (4)	C22—C21—H21	120.2
N3—C11—H11A	108.6	C20—C21—H21	120.2
C12—C11—H11A	108.6	C23—C22—C21	120.3 (4)
N3—C11—H11B	108.6	C23—C22—H22	119.8
C12—C11—H11B	108.6	C21—C22—H22	119.8
H11A—C11—H11B	107.6	C22—C23—C24	119.7 (4)
C11—C12—H12A	109.5	C22—C23—H23	120.1
C11—C12—H12B	109.5	C24—C23—H23	120.1
H12A—C12—H12B	109.5	C25—C24—C23	120.3 (4)
C11—C12—H12C	109.5	C25—C24—H24	119.9
H12A—C12—H12C	109.5	C23—C24—H24	119.9
H12B—C12—H12C	109.5	C20—C25—C24	119.5 (3)
C14—C13—N3	114.5 (7)	C20—C25—H25	120.2
C14—C13—H13A	108.6	C24—C25—H25	120.2
N3—C13—H13A	108.6	C8—N1—C7	122.9 (2)
C14—C13—H13B	108.6	C8—N1—C1	120.9 (3)
N3—C13—H13B	108.6	C7—N1—C1	115.3 (3)
H13A—C13—H13B	107.6	C8—N2—C10	115.6 (3)
N3—C13'—C14'	102.3 (7)	C8—N3—C13'	121.0 (6)
N3—C13'—H13C	111.3	C8—N3—C11	119.2 (4)
C14'—C13'—H13C	111.3	C13'—N3—C11	102.3 (6)
N3—C13'—H13D	111.3	C8—N3—C13	108.4 (4)
C14'—C13'—H13D	111.3	C13'—N3—C13	24.6 (4)
H13C—C13'—H13D	109.2	C11—N3—C13	125.1 (5)
C13'—C14'—H14D	109.5	C16—N4—C9	107.4 (3)
C13'—C14'—H14E	109.5	C16—N4—C20	124.0 (2)
H14D—C14'—H14E	109.5	C9—N4—C20	128.5 (3)
C13'—C14'—H14F	109.5	C17—O3—C18	120.2 (7)
H14D—C14'—H14F	109.5	C17—O3—C18'	111.1 (9)
H14E—C14'—H14F	109.5	C18—O3—C18'	18.0 (13)
C16—C15—C10	105.6 (3)		
			
C6—C1—C2—C3	2.1 (6)	C2—C1—N1—C8	119.2 (4)
N1—C1—C2—C3	178.5 (4)	C6—C1—N1—C8	−64.3 (4)
C1—C2—C3—C4	−0.1 (7)	C2—C1—N1—C7	−71.1 (4)
C2—C3—C4—C5	−1.5 (8)	C6—C1—N1—C7	105.4 (3)
C3—C4—C5—C6	1.2 (7)	N3—C8—N2—C10	−178.4 (3)
C4—C5—C6—C1	0.7 (6)	N1—C8—N2—C10	2.9 (5)
C2—C1—C6—C5	−2.4 (5)	C9—C10—N2—C8	1.3 (5)
N1—C1—C6—C5	−178.9 (3)	C15—C10—N2—C8	175.7 (3)
O1—C7—C9—C10	−177.6 (3)	N2—C8—N3—C13'	−3.7 (8)
N1—C7—C9—C10	0.3 (4)	N1—C8—N3—C13'	175.1 (6)
O1—C7—C9—N4	5.3 (6)	N2—C8—N3—C11	124.5 (4)
N1—C7—C9—N4	−176.9 (3)	N1—C8—N3—C11	−56.7 (5)
N4—C9—C10—N2	174.7 (3)	N2—C8—N3—C13	−27.1 (7)
C7—C9—C10—N2	−3.0 (5)	N1—C8—N3—C13	151.7 (6)
N4—C9—C10—C15	−0.7 (3)	C14'—C13'—N3—C8	−86.6 (10)
C7—C9—C10—C15	−178.3 (3)	C14'—C13'—N3—C11	138.0 (8)
N2—C10—C15—C16	−174.1 (3)	C14'—C13'—N3—C13	−21.7 (15)
C9—C10—C15—C16	0.9 (4)	C12—C11—N3—C8	134.4 (4)
N2—C10—C15—C17	4.2 (6)	C12—C11—N3—C13'	−89.2 (7)
C9—C10—C15—C17	179.2 (3)	C12—C11—N3—C13	−79.0 (8)
C10—C15—C16—N4	−0.9 (4)	C14—C13—N3—C8	−160.6 (8)
C17—C15—C16—N4	−179.3 (3)	C14—C13—N3—C13'	74.4 (19)
C16—C15—C17—O2	176.5 (4)	C14—C13—N3—C11	49.9 (12)
C10—C15—C17—O2	−1.4 (6)	C15—C16—N4—C9	0.5 (4)
C16—C15—C17—O3	−3.0 (5)	C15—C16—N4—C20	176.8 (3)
C10—C15—C17—O3	179.0 (3)	C10—C9—N4—C16	0.1 (4)
C25—C20—C21—C22	−0.5 (5)	C7—C9—N4—C16	177.6 (3)
N4—C20—C21—C22	177.9 (3)	C10—C9—N4—C20	−175.9 (3)
C20—C21—C22—C23	0.4 (6)	C7—C9—N4—C20	1.5 (6)
C21—C22—C23—C24	−0.7 (6)	C25—C20—N4—C16	124.3 (3)
C22—C23—C24—C25	1.0 (6)	C21—C20—N4—C16	−54.1 (5)
C21—C20—C25—C24	0.8 (5)	C25—C20—N4—C9	−60.2 (5)
N4—C20—C25—C24	−177.5 (3)	C21—C20—N4—C9	121.4 (4)
C23—C24—C25—C20	−1.1 (5)	O2—C17—O3—C18	−9.1 (8)
N2—C8—N1—C7	−5.8 (5)	C15—C17—O3—C18	170.5 (6)
N3—C8—N1—C7	175.4 (3)	O2—C17—O3—C18'	8.2 (12)
N2—C8—N1—C1	163.1 (3)	C15—C17—O3—C18'	−172.2 (11)
N3—C8—N1—C1	−15.6 (5)	C19—C18—O3—C17	93.6 (10)
O1—C7—N1—C8	−178.2 (3)	C19—C18—O3—C18'	30 (3)
C9—C7—N1—C8	3.8 (4)	C19'—C18'—O3—C17	144.4 (15)
O1—C7—N1—C1	12.3 (4)	C19'—C18'—O3—C18	−92 (4)
C9—C7—N1—C1	−165.7 (3)		

### Hydrogen-bond geometry (Å, °)

**Table:**

*D*—H···*A*	*D*—H	H···*A*	*D*···*A*	*D*—H···*A*
C6—H6···O1^i^	0.93	2.50	3.320 (5)	147.
C19—H19B···O1^ii^	0.96	2.59	3.545 (8)	175.
C25—H25···O2^iii^	0.93	2.60	3.295 (5)	132.

Symmetry codes: (i) *x*, −*y*+1, *z*−1/2; (ii) *x*−1/2, *y*−1/2, *z*−1; (iii) *x*+1/2, −*y*+1/2, *z*+1/2.
